# Fluorescent enzyme-coupled activity assay for phenylalanine ammonia-lyases

**DOI:** 10.1038/s41598-020-75474-y

**Published:** 2020-10-28

**Authors:** Mădălina E. Moisă, Diana A. Amariei, Emma Z. A. Nagy, Nóra Szarvas, Monica I. Toșa, Csaba Paizs, László C. Bencze

**Affiliations:** Biocatalysis and Biotransformations Research Center, Faculty of Chemistry and Chemical Engineering, Babeș-Bolyai University, Arany János Str. 11, 400028 Cluj-Napoca, Romania

**Keywords:** Biotechnology, Biocatalysis, Analytical biochemistry, Biochemistry, Biocatalysis

## Abstract

Phenylalanine ammonia-lyases (PALs) catalyse the non-oxidative deamination of l-phenylalanine to *trans*-cinnamic acid, while in the presence of high ammonia concentration the reverse reaction occurs. PALs have been intensively studied, however, their industrial applications for amino acids synthesis remained limited, mainly due to their decreased operational stability or limited substrate specificity. The application of extensive directed evolution procedures to improve their stability, activity or selectivity, is hindered by the lack of reliable activity assays allowing facile screening of PAL-activity within large-sized mutant libraries. Herein, we describe the development of an enzyme-coupled fluorescent assay applicable for PAL-activity screens at whole cell level, involving decarboxylation of *trans*-cinnamic acid (the product of the PAL reaction) by ferulic acid decarboxylase (FDC1) and a photochemical reaction of the produced styrene with a diaryltetrazole, that generates a detectable, fluorescent pyrazoline product. The general applicability of the fluorescent assay for PALs of different origin, as well as its versatility for the detection of tyrosine ammonia-lyase (TAL) activity have been also demonstrated. Accordingly, the developed procedure provides a facile tool for the efficient activity screens of large mutant libraries of PALs in presence of non-natural substrates of interest, being essential for the substrate-specificity modifications/tailoring of PALs through directed evolution-based protein engineering.

## Introduction

**Figure 1:**
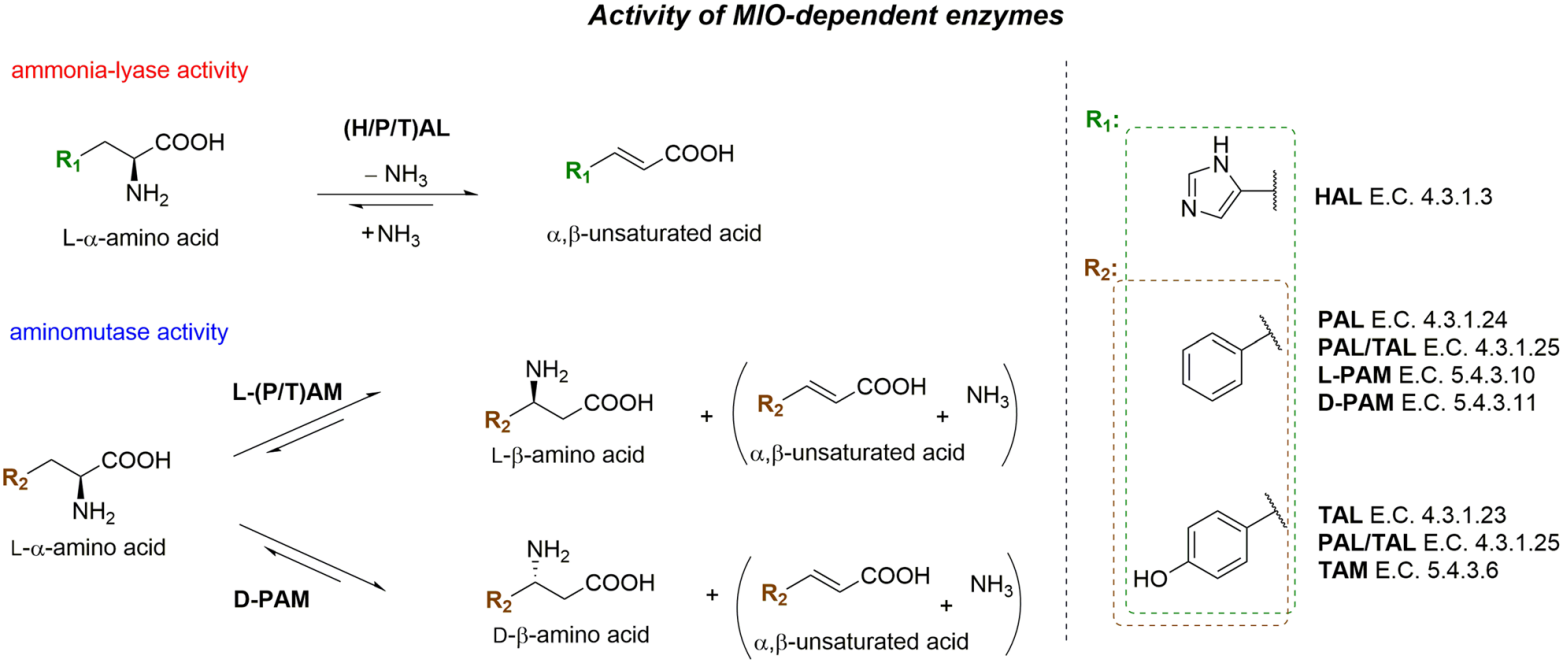
Activities of MIO-dependent enzymes, including aromatic ammonia-lyases and aminomutases.

Phenylalanine ammonia-lyases (PALs, EC 4.3.1.24 and PAL/TALs with dual phenylalanine and tyrosine ammonia-lyase activities, EC 4.3.1.25) catalyze the non-oxidative ammonia elimination of l-phenylalanine to *trans*-cinnamic acid, the precursor for the biosynthesis of phenylpropanoids, such as lignins, flavonoids or coumarins. PALs, similarly to histidine- (HALs E.C. 4.3.1.3) and tyrosine ammonia-lyases (PALs/TALs E.C. 4.3.1.25 and TALs E.C. 4.3.1.23), as well as the corresponding phenylalanine and tyrosine aminomutases (PAMs E.C. 5.4.3.10/11 and TAMs, E.C. 5.4.3.6) (Fig. 1) are MIO-dependent enzymes, all sharing a common 4-methylideneimidazol-5-one (MIO) prosthetic group actively involved in the formation of the reaction intermediate^1–5^. While, under natural conditions, the aromatic ammonia-lyases catalyse the ammonia elimination reaction, in presence of high ammonia concentration they perform the reverse ammonia addition onto α,β-unsaturated arylacrylates, to produce l-α-amino acids in high enantiomeric purity (Fig. 1). Among the most studied aromatic ammonia-lyases PALs from *Rhodotorula* sp. (*Rg*PAL)^6–8^, *Petroselinum crispum* (*Pc*PAL)^9–11^ and *Anabaena variabilis* (*Av*PAL)^12–14^ were shown to display activity on a broad range of substrates. Accordingly, PALs emerged as efficient biocatalysts for the production of d- and l-phenylalanine analogues^2,3,15^, PAL-based industrial processes such as the multi-ton scale production of (*S*)-2,3-dihydro-1*H*-indole-2-carboxylic acid by DSM (Netherlands) being also established^16^. Recent protein engineering of PALs, but also of TALs and PAMs increased their catalytic activity^17–20^, selectivity^21,22^ and/or extended their substrate scope^23–25^. Nonetheless, the application of aromatic ammonia-lyases within the synthetically attractive reverse ammonia addition reaction, for the large scale synthesis of various specific target molecules is still challenging, especially due to occurrence of substrate/product inhibition or enzyme stability issues caused by the high (4–6 M) ammonia concentrations employed within the reaction^18,26,27^. For developing P(T)ALs and P(T)AMs with high operational stability and broad substrate scope novel MIO-enzymes from bacterial (PALs from *Nostoc punctiforme*^13^*, Kangiella koreensis*^28^ and *Planctomyces brasiliensis*^29^, PAL/TAL and PAM from *Pseudomonas fluorescens*^30^), and/or extremophile sources (PALs from *Rubrobacter xylanophilus*^31^ and *Pseudozyma antarctica*^32^) have been explored, some of them with increased thermal- and ionic strength-stability. However, their substrate scope exploration still remains a tedious and laborious task.

Directed evolution driven protein engineering is the most established tool for extending the substrate domain of enzymes, or for increasing their catalytic activity, but also to facilitate protein stability and substrate or product inhibition issues^33–35^. However for the proper selection of hits from the largely sized mutant libraries (> 10^3^–10^4^ clones), high-throughput activity assays are required. In case of PAL mediated reactions, the HPLC methods allowing the determination of conversion values, are suitable for the PAL-activity screens of small sized, focused libraries derived from rational design or single site-CAST (combinatorial active site test)-ing^36^. While high-throughput PAL-activity assays have been developed, their general applicability is still limited. The UV-spectroscopy-based PAL/TAL-activity assay^37,38^ monitoring the production or consumption of the cinnamic acid derivatives, possesses low sensitivity when employed for whole cell- or cell lysate-biocatalysts. Despite our recent improvements of the UV-assay providing applicability for the high-throughput activity screens of saturation mutagenesis libraries^39^, in case of several non-natural substrates, the substrate and product UV–Vis absorption signals, frequently also their maxima, significantly overlap, hindering the assay’s general applicability. The reported fluorescence PAL-activity assays are limited to the use of non-natural substrates, such as *o*-NH_2_-Phe^40^, poorly transformed by PALs or to the detection of PALs with reverse, very uncommon, d-selectivity^14^. Furthermore, the recently developed, high-throughput PAL-activity screening method, based on inductively coupled plasma–mass spectrometr*y*^41^ is also limited by the instrumental requirements.

**Figure 2:**
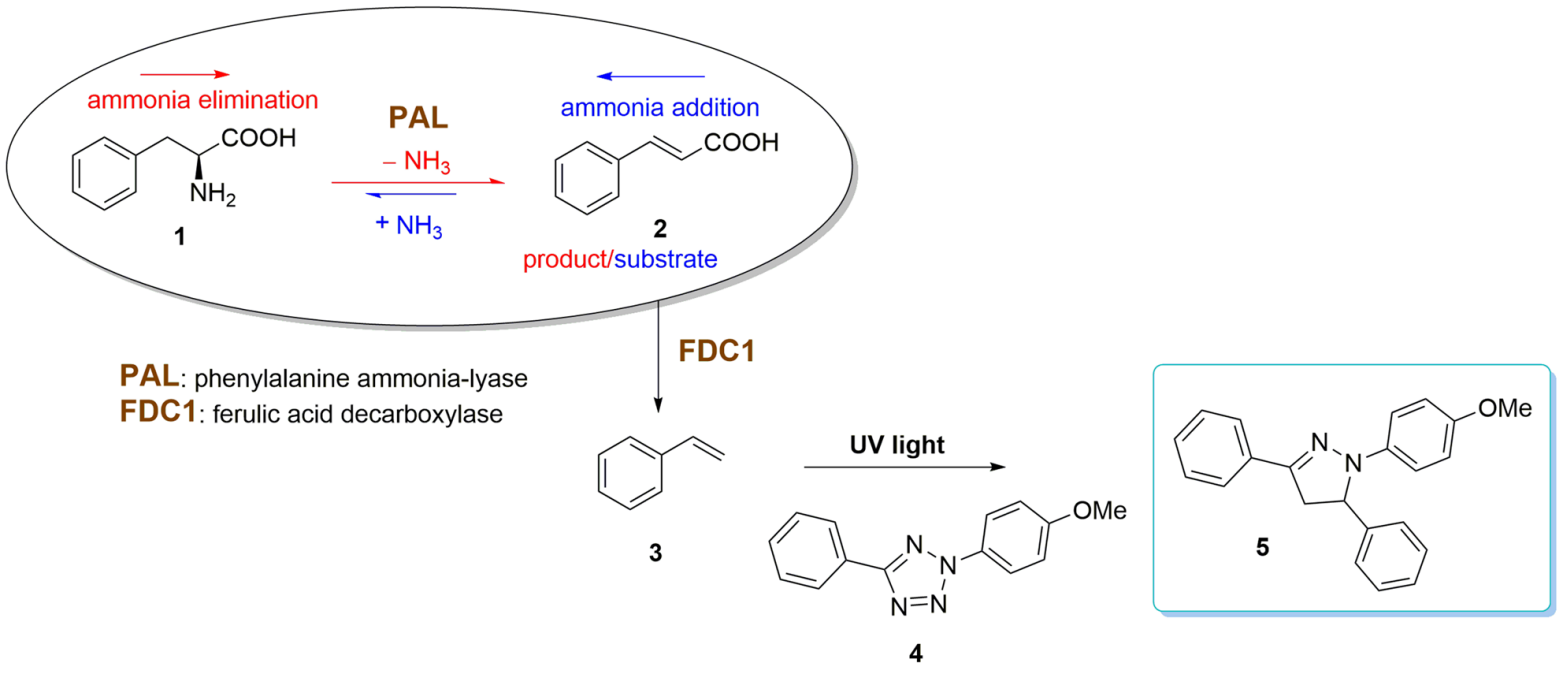
The fluorescent coupled-enzyme assay for PAL-activity assessments.

Herein we report a novel high-throughput fluorescent, enzyme-coupled activity assay for PALs, using ferulic acid decarboxylase (FDC1) to decarboxylate cinnamic acid **2**, the product/substrate of the natural/reverse PAL-reaction, followed by the fluorogenic detection of the produced styrene **3** through its reaction with a tetrazole fluoroprobe **4** (Fig. 2). FDC1 from *Saccharomyces cerevisiae* has already been shown to transform a wide range of phenylacrylate analogues^42,43^ supporting the applicability of the assay for activity screens towards various non-natural substrates of interest. Nonetheless, the sequential use of PAL and FDC1 reactions in genetically engineered whole cells was also demonstrated within the biosynthesis of styrene, 1,2-ethanediols or other valuable products^44–46^. The 1,3-dipolar cycloaddition reaction between an alkene and a tetrazole represents an attractive method of fluorophore-forming bioorthogonal chemistry, with various diaryltetrazoles shown to be highly sensitive fluoroprobes for the detection of alkenes^47–49^.

## Results and discussion

### Fluorogenic reaction set-up

The 1,3-dipolar cycloaddition reaction between an alkene and a nitrile imine, with formation of a fluorescent pyrazoline product, can be conducted either by employing hydrazonoyl chlorides or tetrazoles to generate the nitrile imine through base-catalysed HCl elimination or photoactivation, respectively^50,51^. We opted for the photoinduced 1,3-dipolar cycloaddition using diaryltetrazole **4** as nitrile imine dipole, since it was shown to possess enhanced reaction rate towards *Z*-alkenes and compatibility for in vivo protein labeling within *E. coli* whole cells^47^. Since the solvent/reaction medium of the 1,3-dipolar cycloaddition significantly influences the emission spectra of pyrazoline product **5**, we focused on the selection of the proper reaction medium for the chemo-enzymatic cascade combining the PAL-, FDC1- and 1,3-cyloaddition reactions. Due to the tedious isolation process of holo-FDC1^52,53^ *E. coli* whole cells harbouring the *Saccharomyces cerevisiae fdc1* gene^42^ were used as cellular biocatalyst for the decarboxylation step. While both PAL- and FDC1- enzymatic reaction steps are performed in aqueous medium, styrene **3** and tetrazole **4** are poorly water-soluble and also triaryl-pyrazolines **5** provide higher fluorescence signal turn-on in neat organic solvents^47^. However, their successful use for in vivo/in vitro protein labeling in phosphate-buffer/acetonitrile 1:1 (*v*/*v*) has also been reported^49,54^. Accordingly, to test which solvent system provides more efficient fluorescence detection, we performed the FDC1-mediated decarboxylation of cinnamic acid **2** in phosphate-buffer**,** followed by addition of equal volumes of organic solvents, with various polarities, as the water-miscible acetonitrile or methanol favoring the solubilization of styrene in the reaction mixture, but also *n*-hexane promoting the extraction of styrene. Further, the photoinduced reaction was triggered after the addition of tetrazole **4** dissolved in dimethyl-sulfoxide (DMSO) or *n*-octane. Best fluorescence signal intensities and signal:noise *ratio* were observed when *n*-hexane was used for styrene extraction (see Supplementary Figs. S2–S4 online) and the *n*-hexane extract—tetrazole **4** solution *v*/*v ratio* was > 5.

**Figure 3:**
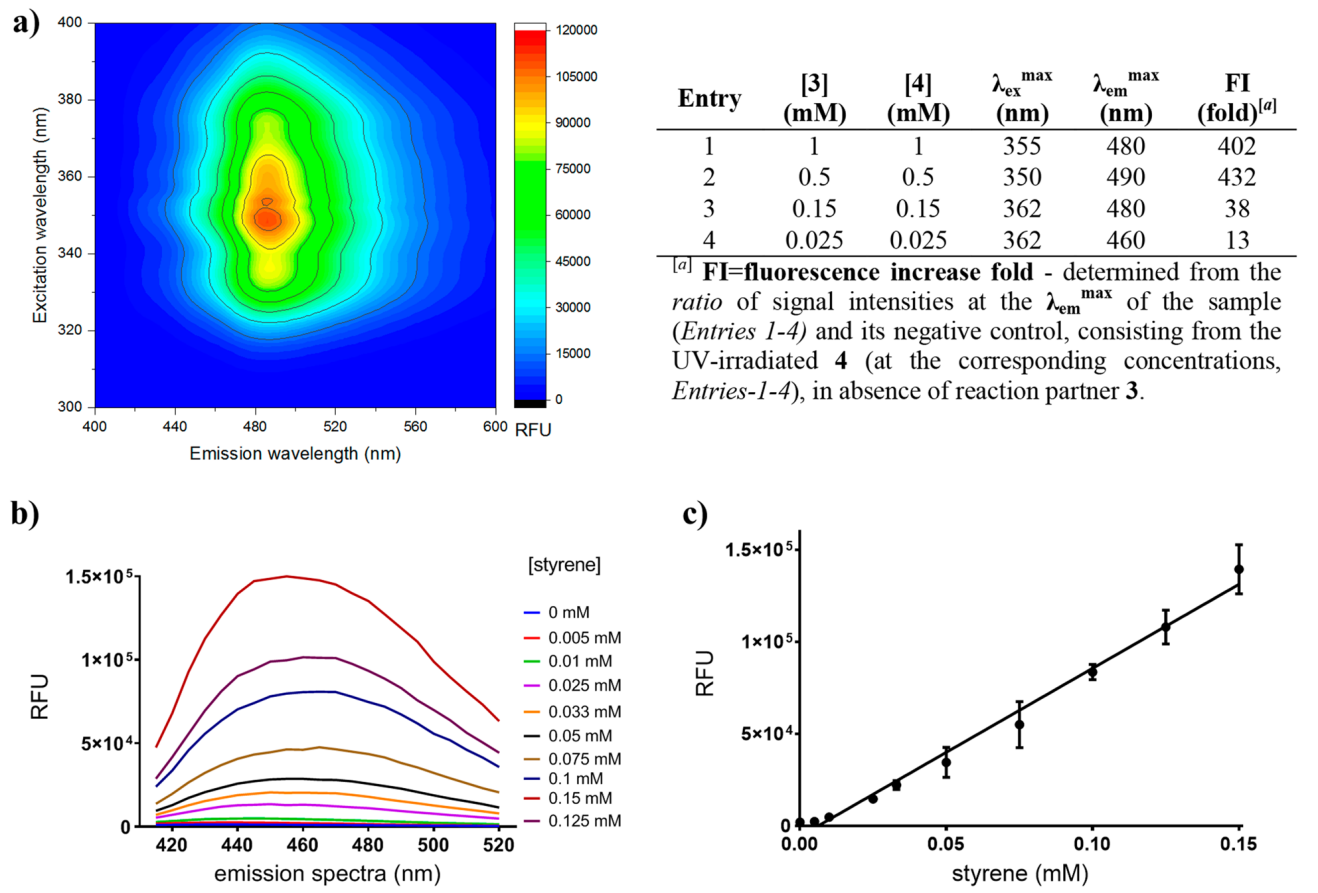
(**a**) Fluorescence signal turn-on at different styrene **3** and fluoroprobe **4** concentrations at the corresponding excitation and emission maxima determined by 3D excitation-emission scans, considering also the background fluorescence (see also Supplementary Fig. S5, S6). (**b**) and **(c**) The detection limit (defined as mean of negative controls plus three times the standard deviation of negative controls) of styrene is 25 μM, while in presence of 0.5 mM of fluorogenic probe **4** linear response occurs for 25 μM-0.1 mM styrene concentration (see also Supplementary Fig. S7).

Next the effect of styrene **3** and tetrazole **4** concentrations on the fluorescence signal turn-on of product **5** were determined by 3D excitation-emission scans (Fig. 3a and Supplementary Fig. S5). Concentrations higher than 0.5 mM of reaction counterparts **3** and **4** provided > 400 fold fluorescence turn-on. Using 0.5 mM tetrazole **4**, a linear response can be obtained at styrene concentrations down to 25 µM and up to 1 mM (Fig. 3b,c and Supplementary Fig. S7). Typical analytical scale PAL reactions are carried out at 0.1–10 mM substrate concentration range. Thus, varying the PAL substrate concentration between 0.5–1 mM range and presuming that the formed cinnamic acid is quantitatively decarboxylated by FDC1, the present protocol allows detection of PAL-activities down to 2.5–5% conversion values, since the linearity of the fluorescence detection is limited by the formation of styrene **3** at least in 25 μM concentration.

### Coupling the PAL-, FDC1- and fluorogenic reactions

Further we tested the coupling of PAL-, FDC1- and fluorogenic reaction steps. Since we targeted the development of PAL-activity assay applicable at whole cell level or cell lysates, suitable for activity assessment of clone-libraries obtained from directed evolution experiments, mixtures of induced *E. coli* whole cells harbouring the recombinant *pcpal* ^55^ and *scfdc1*^42^ genes were tested as catalysts for the transformation of l-Phe into styrene.

First the performance of methods based on the simultaneous or the sequential addition of the two PAL- and FDC1-whole cell biocatalysts was investigated. During the simultaneous procedure both PAL- and FDC1-whole cells were added at the beginning into the reaction mixture, which was then incubated for 16 h, followed by the *n*-hexane extraction and the cycloaddition step. In the sequential procedure, first the PAL-reaction was incubated for 12 h, followed by the addition of FDC1-whole cells and after perfection of the decarboxylation reaction for 4 h, sufficient for the complete conversion of cinnamic acid^42^, similar extraction, cycloaddition steps were performed. In order to rule out the eventuality of unwished cell penetration issues for substrates, intermediates or products, besides the reactions carried out with whole cell-biocatalysts, both procedures were also tested with cell lysates of the induced PAL- and FDC1-whole cells. Important to note, that all protocols were carried out in 96-well-microtiter plates to ensure the high-throughput applicability of the procedure.

**Figure 4:**
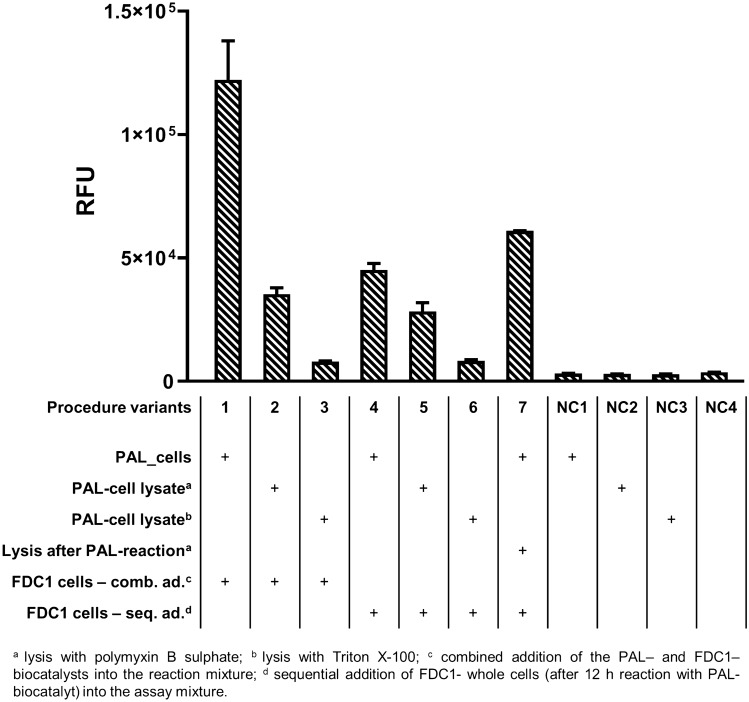
Fluorescent signal intensities for the differently coupled PAL-, FDC1-reactions.

The highest signal intensities were obtained by the simultaneous use of the two PAL- and FDC1- whole cell biocatalysts (Fig. 4, *column 1*), overwhelming those from their sequential addition (Fig. 4, *column 4*), but also those based on the use of cell lysates (Fig. 4, *columns 2, 3, 5, 6*). Moreover, the higher intensities obtained by whole-cell biocatalysts compared to those obtained with cell lysates, suggest that no cell penetration issues occur for compounds **1** and **2**. Supposedly, the incomplete cell-lysis, commonly appearing within microplates, decreases the available enzymatic content of lysates and hence the activity, thus also contributes to the lower signal intensities of these protocols.

To exclude the unfavorable impact of using two separate whole cell-systems for PAL and FDC1 biocatalysts upon the efficiency of the activity assay, the genes encoding *Pc*PAL and *Sc*FDC1 were sub-cloned into pCDFDuet-1 vector, for their co-expression, providing a combined PAL-FDC1 whole cell-biocatalyst, which afforded similar results with that using two individual PAL- and FDC1 whole cell-biocatalysts (see Supplementary Fig. S8). Further, by performing the coupled whole-cell reactions with different batches of induced PAL- and FDC1-whole cells, highly reproducible signal intensities were obtained (see Supplementary Fig. S8).

### Effect of pH

**Figure 5:**
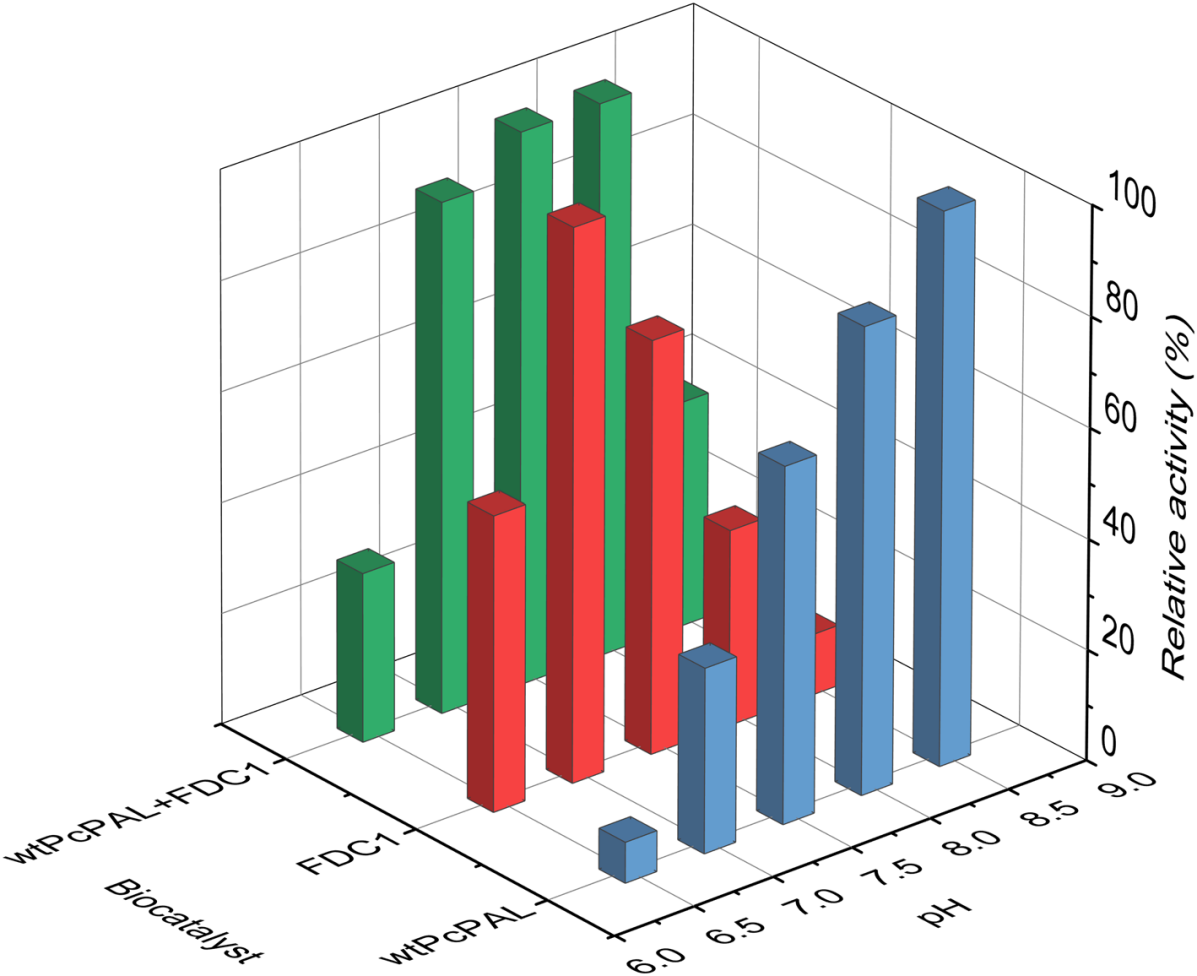
The relative signal intensities of the combined enzyme assay upon performing the PAL- FDC1- whole cell biotransformations at different pH values in comparison with the activity-pH profile of the isolated *wild-type Pc*PAL- enzyme and of the FDC1-whole cell biotransformations.

Since the pH profile of the two enzymes significantly differs, with pH optimums at 6.5–7 for the FDC1 mediated decarboxylation and 8.0–8.5 for the PAL catalysed ammonia elimination, we tested the effect of pH upon the fluorescence signal intensities for the combined PAL-FDC1 reactions. Highest signal intensities were obtained when the enzymatic steps of the assay were performed at pH 7.5–8.0 (Fig. 5).

### Effect of cellular biocatalysts densities

**Figure 6:**
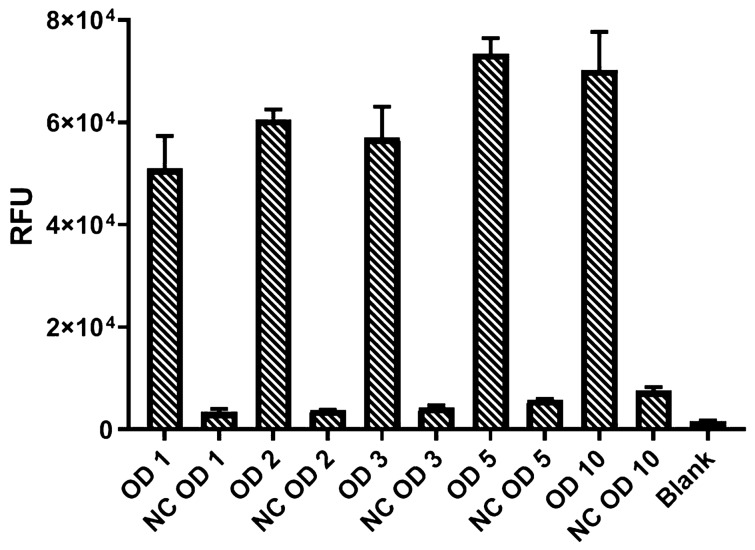
Testing different cellular densities ranging from OD_600_ ~ 1–10 of the FDC1 whole cell-biocatalyst within the combined PAL-FDC1 fluorescent activity assay. The corresponding negative controls (NC) were obtained under similar assay conditions, but without using *Pc*PAL whole cells.

Next, the effect of biocatalysts cell densities upon the fluorescence signal intensities for the combined PAL-FDC1 reactions was studied, working at the optimal pH of 8.0. Since up to this point the reactions were conducted with cell densities of OD_600_ ~ 1 for both *Pc*PAL-and FDC1-whole cell biocatalysts, the concomitant increase of cell densities of both biocatalysts was initially tested. However, since a PAL-assay for high-throughput activity screens is aimed, the increase of cell densities of PAL-biocatalysts is limited by the time necessary for their cellular growth in the microplate format. Shorter assays being favoured, we opted for a ~ 12 h growth of the induced cells within deep-well microplates, resulting in PAL-cell densities of OD_600_ ~ 2. The increase of cell densities for PAL-biocatalysts through cell growth in higher culture volumes, followed by their harvesting and resuspension in several fold lower reaction volumes, resulting thus higher cellular densities, is also hindered by the relatively high optimal assay volume (800 µL) compared to the maximal working-volume of 96-well microplates disposable for cell culturing (~ 1.6 mL for 2.2 mL deep-well plates). Therefore the assay was tested using PAL-biocatalysts with two final OD_600_ of ~ 1 and ~ 2, observing no significant difference in the obtained signal intensities (see Supplementary Fig. S14). However, for the FDC1 whole cells there are no such limitations, since during the desired activity screens of mutant PAL libraries, a previously prepared FDC1 whole cell-biocatalyst can be added into each microplate-well, containing the different variants of PAL-biocatalyst. Thus, performing the growth of FDC1-biocatalyst distinctly, in large fermentation volumes, followed by their aliquoting by harvesting and resuspension in controlled volumes of reaction medium, provides its desired high cell densities within the assay. Accordingly, under constant *Pc*PAL whole cell-concentrations of OD_600_ ~ 1, the rising concentration of the FDC1-biocatalyst increased the fluorescence signal intensities, the highest value being reached at OD_600_ ~ 5 (Fig. 6). Higher FDC1-cell density of OD_600_ ~ 10 didn’t provide further improvements of the protocol, while further cell density increase resulted in highly viscous reaction medium, hindering the assay implementation.

### Method validation

To assess the potential applicability of the assay for directed evolution processes, we tested whether the procedure is suitable to distinguish PALs with different activities towards non-natural substrates. We selected *p*-methoxy-phenylalanine as a model substrate analogue, for which we possess several mutant *Pc*PALs with high activity^18,25^, compared to the *wild-type* enzyme, which has poor activity towards this substrate. Accordingly, the optimized activity assay was performed using induced whole-cells of *Pc*PAL variants F137V, I460V and I460A, known for their high activity towards *p*-MeO-Phe, respectively the poorly active L138V and *wild-type Pc*PAL, and as negative control the reaction without PAL-whole cells.

**Figure 7:**
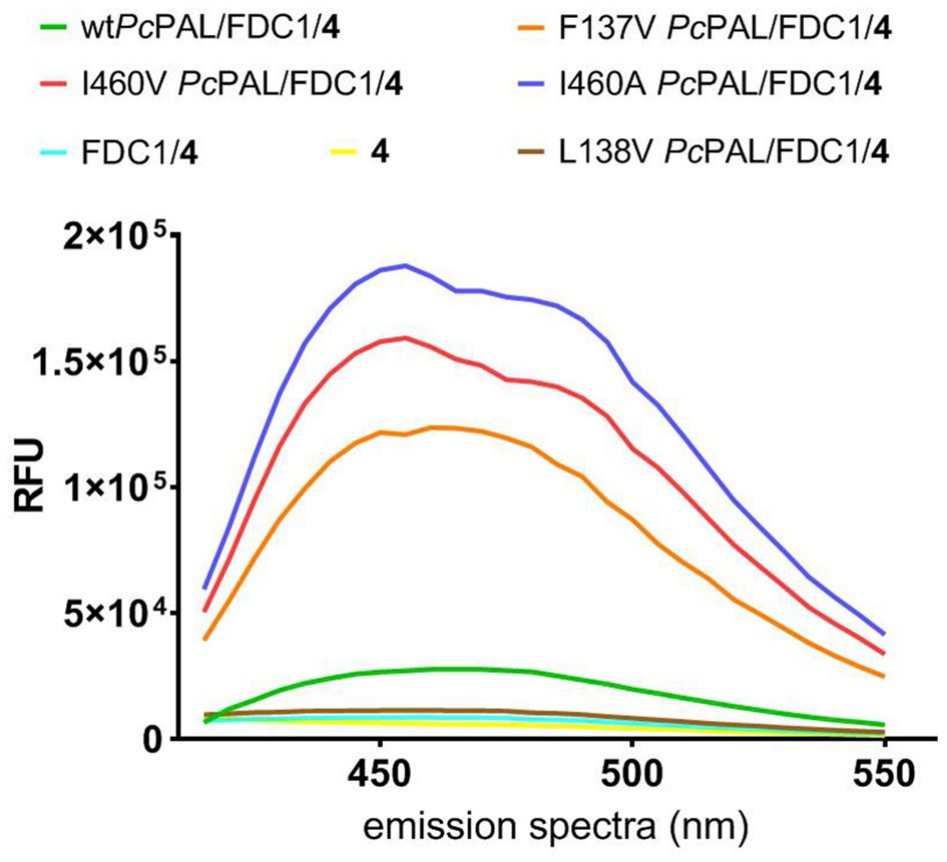
Fluorescence signal intensities obtained by employing the optimized activity assay for the detection of PAL-activity within the ammonia elimination reaction of model substrate, *p*-MeO-Phe.

The highest fluorescence signal intensities, accordingly the highest PAL-activities were recorded for I460V, I460A and F137V variants, clearly overwhelming the signal intensities obtained for the *wild-type Pc*PAL or those of the negative controls, using the inactive L138V variant or reactions without biocatalysts (Fig. 7). The obtained results clearly resemble the activity order based on the conversion values after 16 h reaction time determined by HPLC protocols (Table 1). Furthermore, the increased activities of the I460V, I460A and F137V variants were also confirmed by the enzyme activity measurements monitoring the production of *p*-MeO-cinnamic acid at 290 nm using purified enzymes as biocatalysts. The somewhat larger differences obtained in this case might be explained by the fact that the fluorescent assay employs whole-cell biocatalysts within a reaction time of 16 h, similar to the reactions monitored by HPLC, while the UV assay measures initial reaction rates, in 10 min reaction times using purified enzymes. Thus in case of *Pc*PAL I460A variant, the low relative activity observed within the UV-assay (Table 1) correlates with the reported instability/decreased activity of the purified form of I460A mutant in comparison with its whole cell-biocatalyst form^25^.

**Table 1:** The relative activity order of different *Pc*PAL-variants obtained by the fluorescence-assay in comparison with their activity order obtained through HPLC- and UV-based activity measurements.

*Pc*PAL variant	Relative activities (%)		
	Fluorescence assay^a^	HPLC assay^b^	UV_290nm_-assay^c^
I460A	100	100	16.2
I460V	84.7	94.8	100
F137V	64.3	89.7	14.0
*wild-type*	14.3	7.7	2.5
L138V	6.0	7.7	<1

^a^Determined from the relative fluorescence signal intensities; ^b^Based on the relative conversions values of ammonia elimination reactions after 16, using whole cell PAL-biocatalysts (see Materials and methods); ^c^Determined by UV_290nm_-assay monitoring the *p*-MeO-cinnamic acid production, using the corresponding purified PAL enzymes (see Materials and methods).

To support the general applicability of the procedure among phenylalanine ammonia-lyases, the assay was successfully performed with whole cells harbouring the gene of another phenylalanine ammonia-lyase, PAL from *Arabidopsis thaliana* (*At*PAL) and the *fdc1* gene^44^ (see Supplementary Fig. S15a). Moreover, considering the significant tyrosine ammonia-lyase (TAL) activity of PALs from *Rhodotorula sp*.^56^, the applicability of the assay for the detection of TAL-activities was demonstrated. High fluorescence signal intensities were obtained for the *Rt*PAL/TAL whole cells, while the assay performed with histidine ammonia-lyase (HAL) from *Pseudomonas fluorescens*^30^, known as inactive towards l-tyrosine, provided signals at the level of negative controls (see Supplementary Fig. S15b). The lower signal intensities obtained for the TAL-activity measurements, in comparison with the PAL-assay using l-Phe, might reflect the reported decreased catalytic efficiency of *Sc*FDC1within the decarboxylation of *p*-coumaric acid^43,57^, the product of the TAL-activity. However, ferulic acid decarboxylase of bacterial origin, e.g. FDC from *Enterobacter* sp. (FDC_*E*s) or phenolic acid decarboxylases (PAD) with high activity towards hydroxylated cinnamic acids^43,58^, can provide further increase of the assay sensitivity for applications involving TAL-activity detection.

These results demonstrate the functionality of the developed assay for identification of PAL variants with activity towards non-natural substrate analogues from collections of mutant variants and also the versatility of the assay for the detection of other PAL-similar functions such as tyrosine ammonia-lyase activities.

## Materials and Methods

### Materials

3D scans were performed on a Shimadzu RF 6000 spectrofluorophotometer. Enzyme activity by UV, fluorescence measurements and OD_600_ determinations were performed using a Tecan Spark 10 M microplate reader. Enzyme activity characterization through conversions was performed by HPLC measurements, using an Agilent Technologies 1200 Series instrument equipped with autosampler. The UV irradiation of assay samples was performed in Corning 96-well Clear Flat Bottom UV-Transparent microplates using a handheld Analytik Jena UV lamp of 302 nm. The fluorescence measurement was carried out in Corning 96-well Black Flat Bottom microplates. Styrene was purchased from Sigma Aldrich and solvents (*n*-hexane, *n*-octane, dimethyl sulfoxide, acetonitrile) of HPLC grade were purchased from VWR. The synthesis of fluorogenic probe **4** was performed according to reported procedure^47^.

### Calibration curve

Styrene **3** solutions in *n*-hexane (0–0.15 mM) were exposed to tetrazole **4** (in a final concentration of 0.5 mM) and irradiated at 302 nm for 1 min with a UV lamp in a Corning 96-well Clear Flat Bottom UV-transparent microplate. The reaction volume was 200 μL. 100 μL from the reaction mixtures were moved into Corning 96-well Black Flat Bottom microplates and the fluorescence measurements were performed setting the excitation wavelength at 360 nm and the emission at 460 nm using TECAN Spark 10 M microplate reader. All experiments were performed in triplicate and standard deviations from mean values are given.

### 3D scans

Samples of 3 mL were prepared as follows: a solution of styrene **3** in *n*-hexane was mixed with the solution of tetrazole **4** in *n*-octane, both in final concentration of 0.025, 0.15, 0.5 or 1 mM, irradiated at 302 nm for 1 min with a handheld UV lamp and immediately analyzed with a spectrophotofluorimeter. The excitation wavelength domain was set to 300–400 nm and the emission wavelength domain was set to 400–600 nm. Negative controls/background samples consisting in the mixture of **3** and **4** solutions without irradiation and **4** solution alone irradiated, as described above, were also measured.

### Cell cultures in 96-deep well microplates

400 µL of sterile LB medium supplemented with carbenicillin (50 μg/mL) and chloramphenicol (34 μg/mL) was inoculated with *Pc*PAL *E. coli* Rosetta (DE3) pLysS cells and incubated overnight at 37 °C and 180 rpm in 96-deep well microplates. 20 µL of this culture was inoculated into 1 mL sterile, fresh LB medium and incubated at 37 °C and 180 rpm until OD_600_ ~ 0.6 was reached (approx. 3 h), when induction was started by the addition of 0.5 mM IPTG and the incubation temperature was set to 30 °C. At OD_600_ ~ 2, cells from 400 µL of culture were harvested by centrifugation at 4000 rpm, 4 °C for 20 min.

### Assay set-up

*Pc*PAL whole cells (OD_600_ ~ 2) were suspended in 400 μL substrate solution (l-Phe, 2 mM) in Tris buffer (20 mM Tris, 100 mM NaCl, pH 8) and 400 μL of FDC1 cells (OD_600_ ~ 2) in Tris buffer were added. In experiments where lysis was tested, the substrate solution was added to the *Pc*PAL cell lysate (obtained as described in protocols below), maintaining the same final substrate concentration (1 mM), followed by lysis after FDC1 reaction. Lysis after the PAL reaction was also investigated. The reactions were incubated overnight at 30 °C and 200 rpm. For reactions performed with sequential addition of the two biocatalysts (with and without lysis), PAL reactions were carried out overnight (approx. 12 h) and the next day FDC1 cells were added and the reactions were stopped after additional 4 h. Next, extractions with 800 µL *n*-hexane were performed, followed by centrifugation at 4000 rpm, 4 °C for 20 min. 175 µL of extract was moved into a Corning 96-well Clear Flat Bottom UV-transparent microplate and mixed with 25 µL of diaryltetrazole 1 mg/mL solution in *n*-octane. The UV reaction was carried out at 302 nm for 1 min using a handheld UV lamp. 100 µL from the samples were moved into Corning 96-well Black Flat Bottom plates and the fluorescence measurement was performed setting the excitation wavelength at 360 nm and the emission at 460 nm using TECAN Spark 10 M microplate reader. Two negative controls prepared identically as the reactions but omitting the *Pc*PAL or the FDC1 cells, were also measured. The blank sample (the background) consisted of 175 µL of *n*-hexane mixed with 25 µL of diaryltetrazole probe (prepared as previously described). The negative controls and the blank sample were irradiated and measured as described above. All experiments were performed in triplicate and standard deviations from mean values are given.

*Lysis with Triton X-100:* cells (from 1 mL culture of OD_600_ ~ 2) were incubated in lysis buffer (50 mM Tris-HCl pH 8.5, 150 mM NaCl, 1 mM EDTA, 1% Triton X-100 *v*/*v*, 1 mg/mL lysozyme) at room temperature for 10 -15 min with occasional mixing. The pellet is next removed by centrifugation at 10,000 rpm for 10 min at 4 °C.

*Lysis with polymyxin B sulphate (PMBS)*: cells (from 1 mL culture of OD_600_ ~ 2) were incubated in lysis buffer (20 mM Tris-HCl pH 8.0, 16 mg/mL lysozyme, 30 mg/mL PMBS) at 45 °C and 200 rpm for 20 min. The pellet was removed by centrifugation at 10,000 rpm for 10 min at 4 °C.

### pH screen for *Pc*PAL-mediated reaction

*Pc*PAL activity (5 µg of isolated enzyme) in buffer solutions (200 µL reaction volume) of different pH values (pH 6.5–8.5) was measured spectrophotometrically by monitoring the production of *trans*-cinnamic acid (290 nm) from l-Phe (2 mM) for 5 min using a Tecan Spark 10 M instrument, in Corning 96-well Clear Flat bottom UV-Transparent microplate, in triplicate.

### pH screen for the coupled FDC1-fluorescent reaction system

FDC1 cells (OD_600_ ~ 1) were resuspended in buffer solutions (784 µL) of different pH values (pH 6.5–8.5) to which *trans*-cinnamic acid was added from a stock solution of 50 mM in DMSO (16 μL) to a final concentration of 1 mM. Reactions performed in 96-deep well microplates were incubated for 3 h at 30 °C and 200 rpm. Extractions, UV irradiation and fluorescence measurements were performed as mentioned above. The blank sample consisting in the tetrazole dissolved in *n*-hexane was included in this experiment, while all experiments were performed in triplicate.

### pH screen for coupled *Pc*PAL-FDC1–fluorescent reaction system

Reactions were performed as previously described for experiments with co-incubation and without lysis, testing buffer solutions with different pH values (pH 6.5–8.5). Extractions, UV irradiation and fluorescence measurement were performed as mentioned above. The blank sample consisting in the tetrazole dissolved in *n*-hexane was included in this experiment, while all experiments were performed in triplicate.

### Effect of biocatalysts cell densities upon signal intensities

Reactions were performed as previously described for experiments with co-incubation and without lysis, in Tris buffer of pH 8.0, testing different FDC1 whole cells concentrations (OD_600_ ~ 1–10). Extractions, UV irradiation and fluorescence measurement were performed as mentioned above. The blank sample was prepared as previously described and the negative controls corresponding to each reaction consisted in reaction mixtures without *Pc*PAL whole cells but containing FDC1 whole cells of OD_600_ ~ 1–10. All experiments were performed in triplicate and standard deviations from mean values are given.

### Biotransformations monitored by HPLC

PAL activity was characterized through conversion values of the ammonia elimination reaction of 4-methoxy-phenylalanine**,** performed at 30 °C in 500 µL reaction volume using 1 mM *rac*-*p*-MeO-Phe in 100 mM Tris-HCl, pH 8.8 and the different whole cell-biocatalysts of *wt-*, I460V, I460A, F137V and L138V *Pc*PAL variants, with a final cell density of OD_600_ ~ 1. The biotransformations were shaken at 200 rpm for 16 h, followed by the removal of 100 µL sample and quenching through addition of an equal volume of MeOH, vortexed, and centrifuged (13,400 rpm/12 000 g, 10 min). The supernatant was filtered through a 0.22 μm nylon membrane filter and analyzed by reverse-phase HPLC, according to previously reported procedures^25^.

### Enzyme activity measurement by UV using isolated enzymes

PAL activity was measured spectrophotometrically in the ammonia elimination reaction of 4-methoxy-phenylalanine using a Tecan Infinite Spark 10 M microplate reader and Corning 96-well Clear Flat Bottom UV-Transparent microplates. Reactions were done in triplicate at 30 °C in a 200 μL reaction volume using 1 mM substrate (in 100 mM Tris-HCl, 120 mM NaCl, pH 8.8) and 5 μg enzyme (*wtPc*PAL, I460V, I460A, F137V, L138V), monitoring the production of *trans*-cinnamic acid analogue through absorbance at 290 nm.

### Final assay conditions

*Pc*PAL-whole cells (OD_600_ ~ 2) grown and harvested as above mentioned were suspended in 400 μL substrate solution (l-Phe or *rac*-*p*-MeO-Phe, 2 mM) in Tris buffer (20 mM Tris, 100 mM NaCl, pH 8) and 400 μL of FDC1 cells (OD_600_ ~ 10) in Tris buffer were added. The reactions were incubated overnight at 30 °C and 200 rpm. Next, extractions with 800 µL *n*-hexane were performed, followed by centrifugation at 4000 rpm, 4 °C for 20 min. 175 µL of extract was moved into a Corning 96-well Clear Flat Bottom UV-transparent microplate and mixed with 25 µL of diaryltetrazole 1 mg/mL solution in *n*-octane. The UV reaction was carried out at 302 nm for 1 min using a handheld UV lamp. 100 µL from the samples were moved into Corning 96-well Black Flat Bottom plates and the fluorescence measurement was performed setting the excitation wavelength at 360 nm and the emission at 460 nm (for l-Phe) or 455 nm (for *rac*-*p*-MeO-Phe) using TECAN Spark 10 M microplate reader. Two negative controls prepared identically as the reactions but omitting the *Pc*PAL or the FDC1 cells, were also measured. The blank sample consisted of 175 µL of *n*-hexane mixed with 25 µL of diaryltetrazole probe (prepared as previously described). The negative controls and the blank sample were irradiated and measured as described above. All experiments were performed in triplicate.

## Conclusions

The study describes the development of a fluorescence enzyme-coupled phenylalanine ammonia-lyase activity assay aimed to provide proper identification/selection of hits from the largely sized mutant libraries obtained through directed evolution processes. The developed assay employs PALs as whole-cell biocatalysts, while the cinnamic acid product of the PAL-reaction is decarboxylated by ferulic acid decarboxylase from *Saccharomyces cerevisiae* (*Sc*FDC1) producing styrene, that can be sensitively detected through fluorophore-forming bioorthogonal chemical procedure, using tetrazole reactants. During the assay set-up several reaction parameters, such as reaction medium, pH, biocatalyst cell density, reactants concentrations, have been optimized in order to obtain the maximal signal:noise *ratio*. The optimal assay conditions were validated using a set of mutant *Pc*PAL variants with different and known orders of activity within the ammonia elimination from the non-natural substrate analogue, *p*-methoxy-phenylalanine, while its general applicability was demonstrated through performing the assay with PALs from different sources, such as *Pc*PAL from *Petroselinum crispum* and *At*PAL from *Arabidopsis thaliana*. Moreover exploiting the tyrosine ammonia-lyase (TAL) activity of *Rt*PAL from *Rhodotorula toruloides*, the applicability of the assay for TAL-activity screens was also demonstrated.

The developed fluorescent activity assay provides a facile tool for the efficient activity screens of mutant libraries of phenylalanine ammonia-lyases within the reactions of non-natural substrates of interest, essential for substrate-specificity modifications/tailoring of PALs through directed evolution based protein engineering.

## Supplementary information

Supplementary Information
