# Supplementary material for: Fluorescent enzyme-coupled activity assay for phenylalanine ammonia-lyases
Source: Sci Rep. 2020 Oct 28;10:18418. doi: 10.1038/s41598-020-75474-y (PMC7595223; doi:10.1038/s41598-020-75474-y)
Supplement: Supplementary file 1 — Supplementary Information [file 41598_2020_75474_MOESM1_ESM.pdf]

Supplementary Information

Fluorescent enzyme-coupled activity assay for phenylalanine ammonia-lyases

Mădălina Elena Moisă, Diana Amariei, Emma Zsófia Aletta Nagy, Nóra Szarvas, Monica Ioana Toşa, Csaba Paizs, László Csaba Bencze*

Biocatalysis and Biotransformations Research Center, Faculty of Chemistry and Chemical Engineering, Babeș-Bolyai University, Arany János Str. 11, RO-400028 Cluj-Napoca, Romania

* Address for correspondence: Dr. László Csaba Bencze, E-mail: cslbencze@chem.ubbcluj.ro

Table of contents

1. Fluorogenic reaction set-up/Solvent optimizations 3

1.1. PBS *versus* PBS/Acetonitrile system 3

1.2. Testing the PBS, PBS/hexane and PBS/methanol systems 4

1.3. PBS/*n*-hexane procedure and negative controls 4

2. 3D excitation and emission scans 5

3. Calibration with styrene 6

4. Assay reproductibility 7

5. Synthesis of the fluorogenic diaryltetrazole probe **4** 7

5.1. The synthesis of phenylsulfonylhydrazone (II) 8

5.2. The synthesis of 4-methoxyphenyldiazonium salt (V) 8

5.3. The synthesis of 2-(4-methoxyphenyl)-5-phenyl-2*H*-tetrazole (III) 8

6. The effect PAL- and FDC1- whole cell densities upon the fluorescent signal intensities 10

7. Method validation 11

8. Construction of pcpal-fdc1-pCDFDuet-1 expression vector 12

9. References 13

1. Fluorogenic reaction set-up/Solvent optimizations

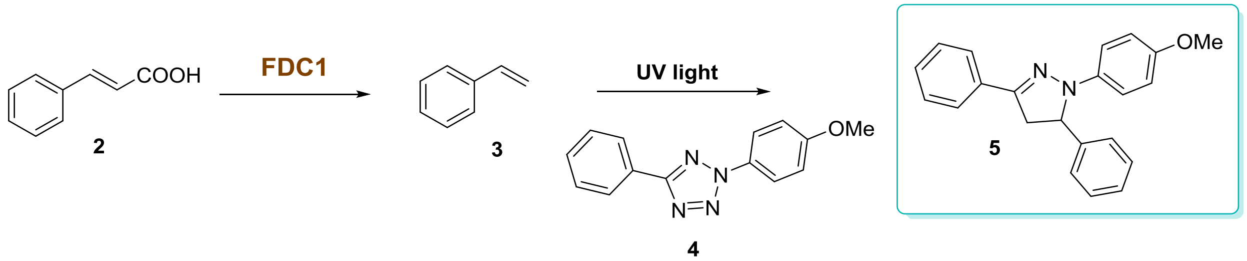

Supplementary Figure S1. Testing/set-up of the fluorescence assay starting from cinnamic acid **2**, the product of the natural PAL-reaction.

1.1. PBS *versus* PBS/Acetonitrile system

Assay conditions: The FDC1-mediated whole cell-biotransformations were performed by incubating the reaction mixture of 0.5 mM cinnamic acid **2** and whole cell-FDC1-biocatalyst of cell density of OD_{600} ~1 in 1 mL PBS buffer (20 mM NaH_2PO_4, 100 mM NaCl, pH 7.5) for 12 h at 200 rpm, 30 ^{0}C. After 12h reaction time, 1 mL MeCN (PBS/MeCN system) or PBS (PBS system) was added to the reaction mixture. To 100 µL sample from the obtained reaction mixture addition of different equivalents (0.01 eq., 0.1 eq. or 1 eq. relatively to the concentration of cinnamic acid **2**) of the fluorogenic probe **4** (from its DMSO stock solution) was performed, followed by irradiation at 302 nm for 1 min and fluorescence measurements.

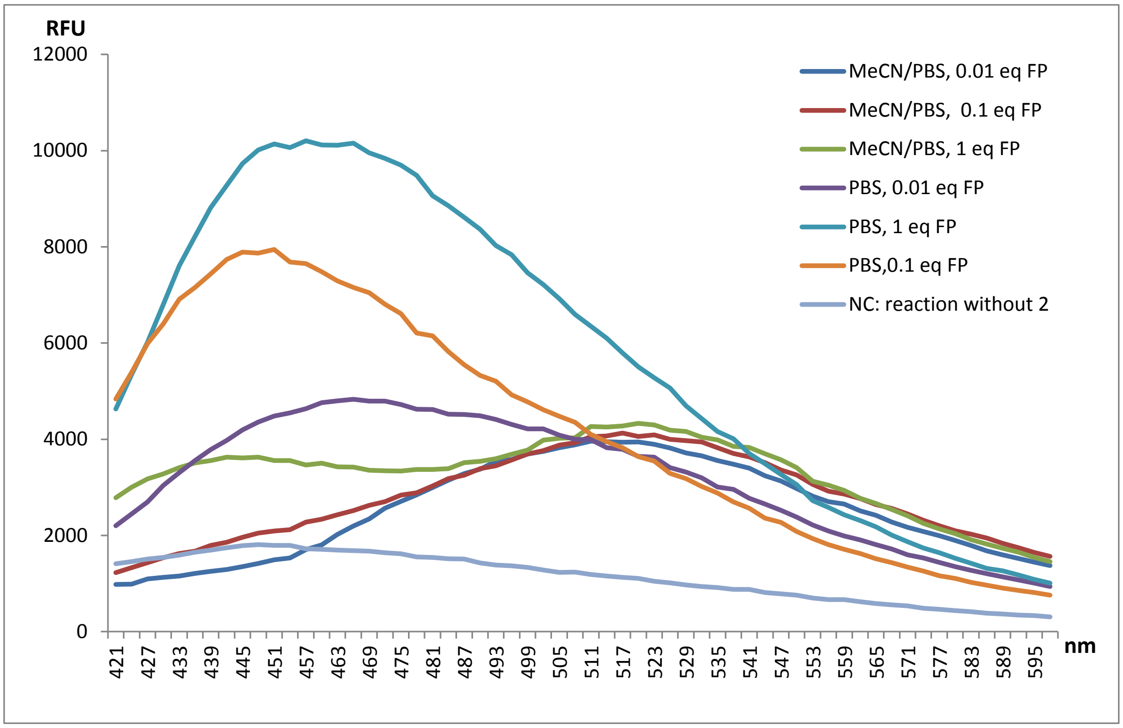

Supplementary Figure S2. Testing different PBS and PBS/MeCN media under different fluoroprobe concentrations for the coupling of the fluorogenic reaction between **3** and **4** with the FDC1-mediated biotransformation of cinnamic acid **2**. The higher signal intensities favour the use of PBS as reaction medium and 1 eq. of fluorogenic probe **4**.

1.2. Testing the PBS, PBS/hexane and PBS/methanol systems

Assay conditions: The FDC1-mediated whole cell-biotransformations were performed by incubating the reaction mixture of 0.5 mM cinnamic acid **2** and whole cell-FDC1-biocatalyst of OD_{600}~1 in 1 mL PBS buffer (20 mM NaH_2PO_4, 100 mM NaCl, pH 7.5) for 12 h at 200 rpm and 30 ^{0}C. After 12h reaction time, *a*) 1 mL methanol was added to the reaction mixture (PBS/methanol) or *b*) extraction with 2 x 1 mL *n*-hexane (PBS/hexane) or *c*) dilution with additional 1 mL PBS was performed (PBS). In other variants of the procedure, in order to test the effect of whole cell-biocatalyst removal, after 12h reaction time a centrifugation step (13000 rpm, 12000 x g) was included and the obtained supernatant (SN_PBS) was processed further with addition of *a*) methanol, *b*) *n*-hexane or *c*) PBS, similarly as described above. Into 100 µL of the obtained mixtures/extracts of the different *a*, *b* and *c* procedure variants, the addition of 50 µL of the fluorogenic probe **4** was added from its stock solutions in *n*-octane (**Fig. S3a**) or DMSO (**Fig. S3b**) for a final concentration of 0.25 mM (corresponding to 1 eq. of **4** relatively to **2**), followed by irradiation at 302 nm for 1 min and fluorescence measurements.

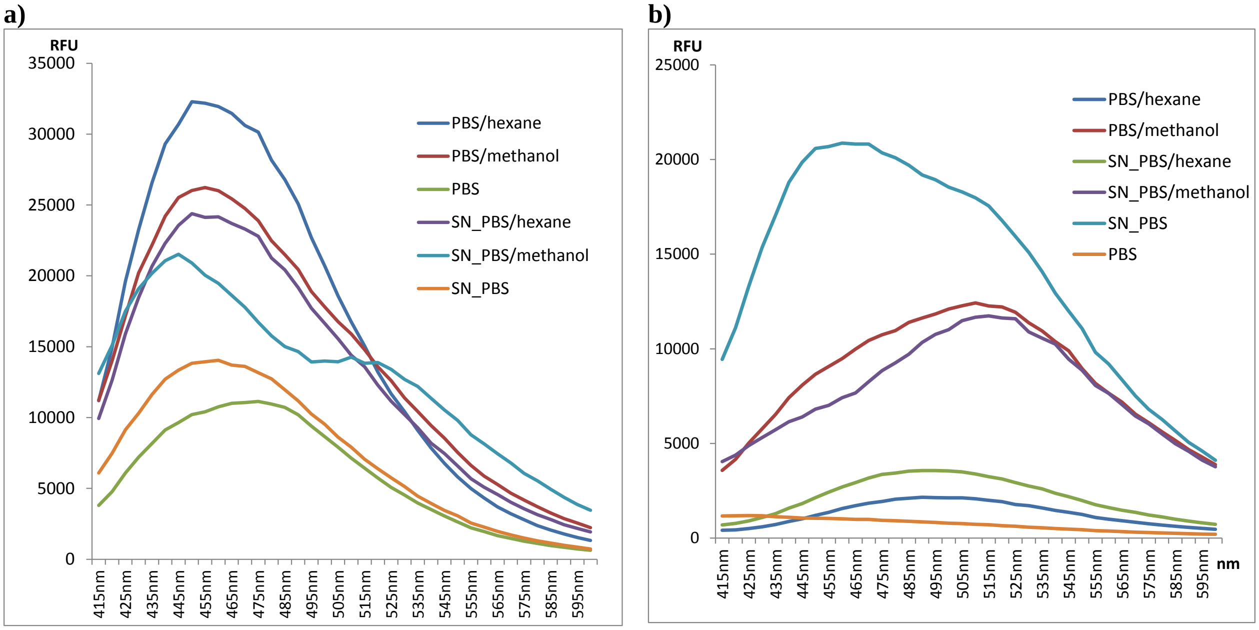

Supplementary Figure S3: Testing the use of PBS, PBS/MeOH and PBS/hexane procedures, as well as the effect of cell removal after the biotransformation step ("SN_"procedures) upon the fluorescent signal intensities, under the use of constant molar *ratio* of 1 for fluorogenic probe **4**: cinnamic acid **2**, adding the fluorogenic probe **4** from its stock solution in **a)** *n*-octane or **b)** DMSO.

1.3. PBS/*n*-hexane procedure and negative controls

Assay conditions: The FDC1-mediated whole cell biotransformations were performed by incubating the reaction mixture of 1 mM cinnamic acid **2** and whole cell-FDC1-biocatalyst of OD_{600}~1 in 1.5 mL PBS buffer (20 mM NaH_2PO_4, 100 mM NaCl, pH 7.5) for 12 h at 200 rpm and 30 ^{0}C. After 12h reaction time the reaction mixture was extracted with 3 x 0.5 mL *n*-hexane. Into the 100 µL of *n*-hexane extract 50 µL of the fluorogenic probe **4** was added from its stock solution-in *n*-octane in final concentration of 1 mM, corresponding to 1 eq. of **4** relatively to **2**, followed by irradiation at 302 nm for 1 min and fluorescence measurements. Negative controls consisted of: *1)* assay performed under similar conditions, adding instead of the fluorogenic probe (FP) **4** solution only *n*-octane (**NC1**); *2)* assay performed with initial solution without substrate, cinnamic acid **2** (**NC2**); 3) assay performed without the decarboxylation step, using 100 µL 1 mM styrene **3** solution in *n*-hexane and in the absence of fluorogenic probe (FP) **4** (**NC3**).

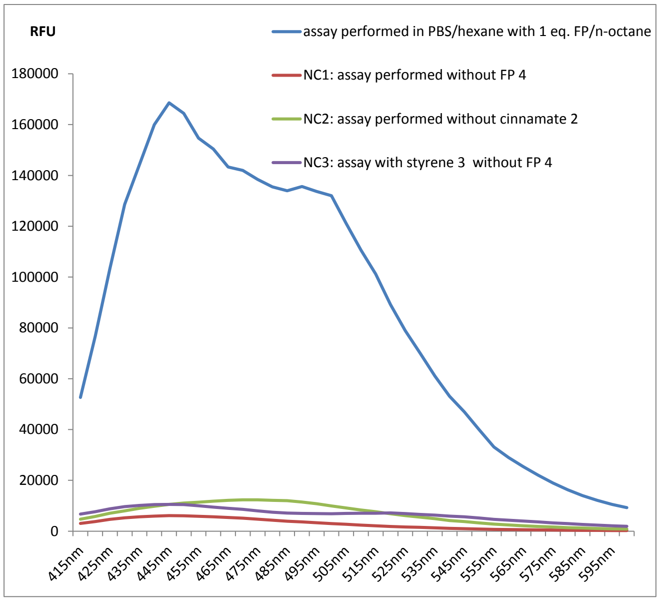

Supplementary Figure S4. Fluorescent signals of the optimal assay condition of the PBS/*n*-hexane procedure in comparison with the corresponding negative controls.

2. 3D excitation and emission scans

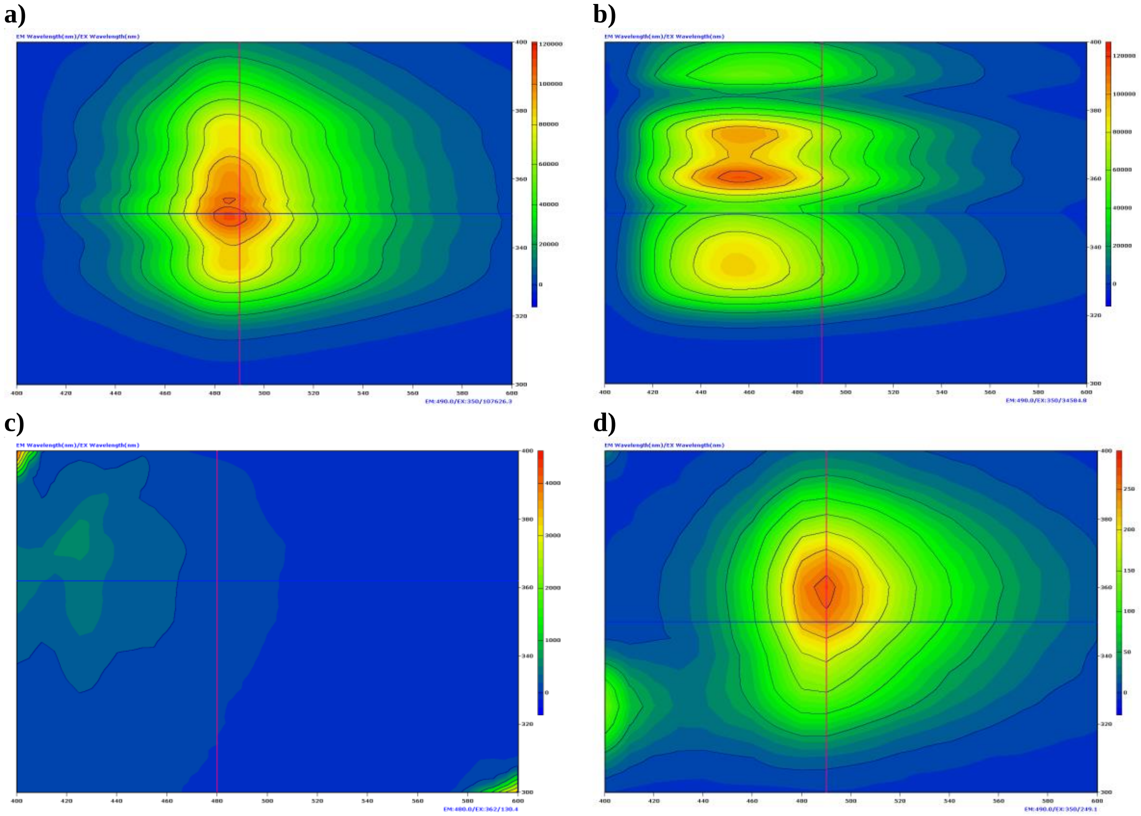

Supplementary Figure S5. 3D scans for **a)** styrene (0.5 mM) and diaryltetrazole (0.5 mM) with UV irradiation; **b)** styrene (0.5 mM) and diaryltetrazole (0.5 mM) without UV irradiation; **c)** styrene (0.5 mM) with UV irradiation; **d)** diaryltetrazole (0.5 mM) with UV irradiation. (For detailed experimental conditions of 3D-scans see main manuscript/Materials and methods)

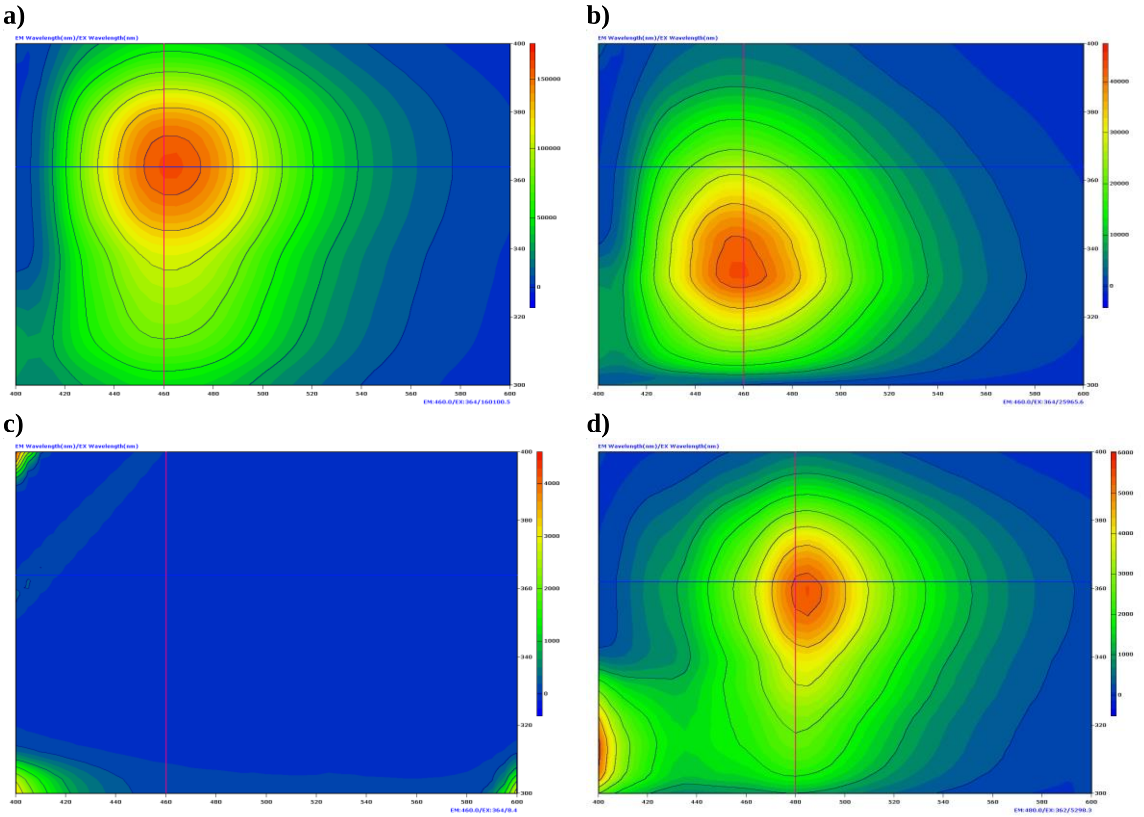

Supplementary Figure S6. 3D scans for **a)** *p*-MeO-styrene (0.5 mM) and diaryltetrazole (0.5 mM) with UV irradiation; **b)** *p*-MeO-styrene (0.5 mM) and diaryltetrazole (0.5 mM) without UV irradiation; **c)** *p*-MeO-styrene (0.5 mM) with UV irradiation; **d)** diaryltetrazole (0.5 mM) with UV irradiation. (For detailed experimental conditions of 3D-scans see main manuscript/Materials and methods)

3. Calibration with styrene

In order to determine the styrene concentration detectable by the fluorescent activity assay calibration with styrene solutions of (0-1 mM in *n*-hexane) and solution of 0.5 mM diaryltetrazole (in *n*-octane) was performed in 200 μL *n*-hexane. The samples were photoirradiated at 302 nm in UV transparent Corning 96-well Clear Flat Bottom UV-transparent plates at room temperature for 1 min. 100 μL from samples was placed into Corning 96-well Black Flat Bottom plates and using 360 nm excitation wavelength the fluorescence emission (410-520 nm) was measured. Using the values obtained at emission maxima of 460 nm, the calibration curves (**Fig. S7**) were obtained. Since at ≥ 0.2 mM styrene concentrations the fluorescent signal intensities exceeded the dynamic range of the instrument, these samples were 100 fold diluted with *n*-hexane, thus two calibration curves were obtained for a) low styrene concentrations (0.005-0.15 mM) – **Fig. S7a** and b) for higher styrene concentrations (0.2-1 mM) – **Fig. S7b**. Negative controls consisted of irradiated solutions containing only 0.5 mM diaryltetrazole **4** in *n*-hexane.

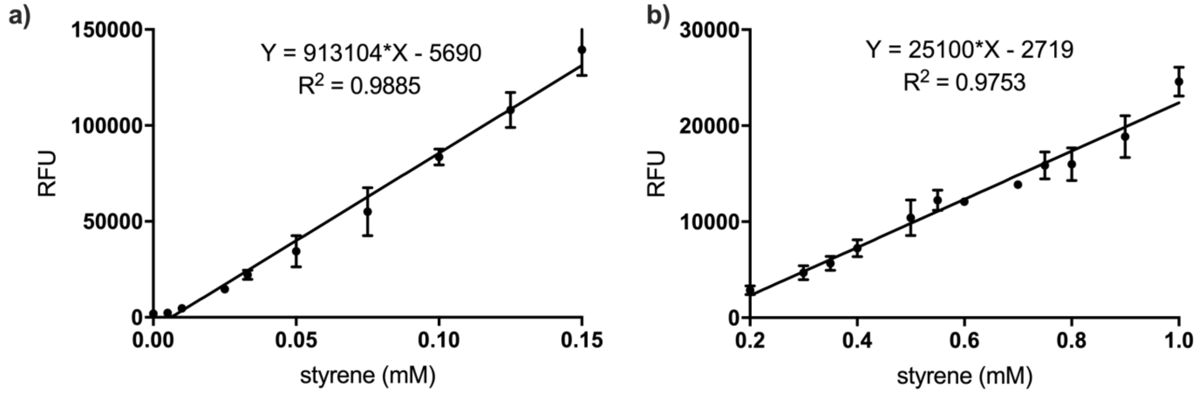

Supplementary Figure S7. Calibration curve for **a)** low styrene concentrations (0.005-0.15 mM) and **b)** high styrene concentrations (0.2-1 mM).

4. Assay reproductibility

Assay conditions: The assay was performed under the optimized conditions described in the Materials and methods of the main manuscript, in one case (**Fig. S8a**) using two separate *E. coli* whole cell-systems for *Pc*PAL and FDC1, while in the other case (**Fig. S8b**) *E. coli* whole cells harbouring pCDFDuet-1 plasmid including the two genes of *Pc*PAL and FDC1 were employed. The combined *Pc*PAL-FDC1 whole cell-biocatalyst was obtained identically as *Pc*PAL whole cell-biocatalyst by cultivation in 96-deep well microplates as described in the main manuscript, section Materials and methods.

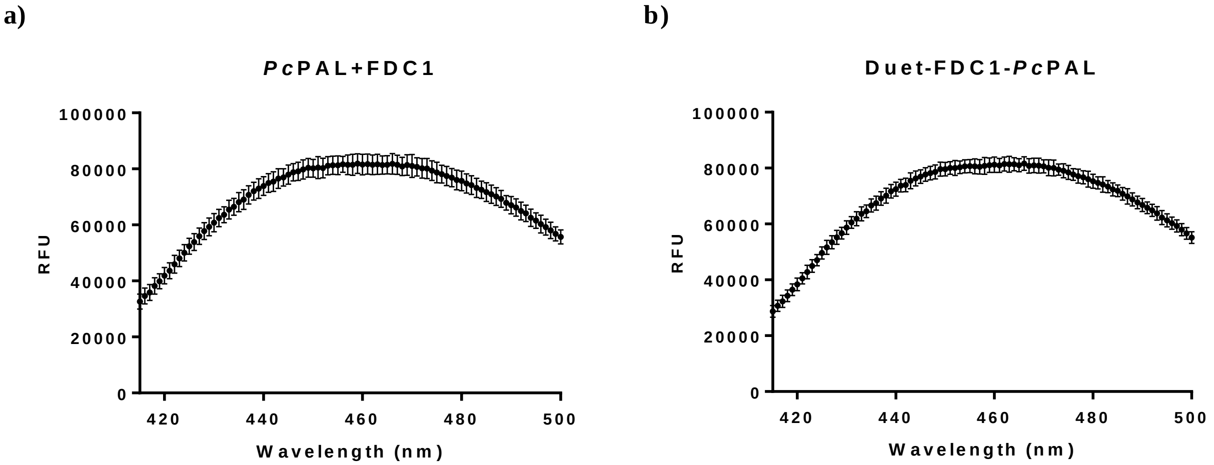

Supplementary Figure S8. Assay reproducibility using *Pc*PAL and FDC1 as **a)** two individual whole cell-biocatalysts and **b)** single, combined whole cell-biocatalyst.

5. Synthesis of the fluorogenic diaryltetrazole probe 4

Starting from the commercially available aldehyde (**I**) through the reaction with phenylsulfonylhydrazine we obtained the phenylsulfonylhydrazone (**II**), followed by its reaction with 4-methoxyphenyldiazonium salt (**V**) in pyridine which afforded the 2-(4-methoxyphenyl)-5-phenyl-2*H*-tetrazole (**III**) (**Fig. S9**).

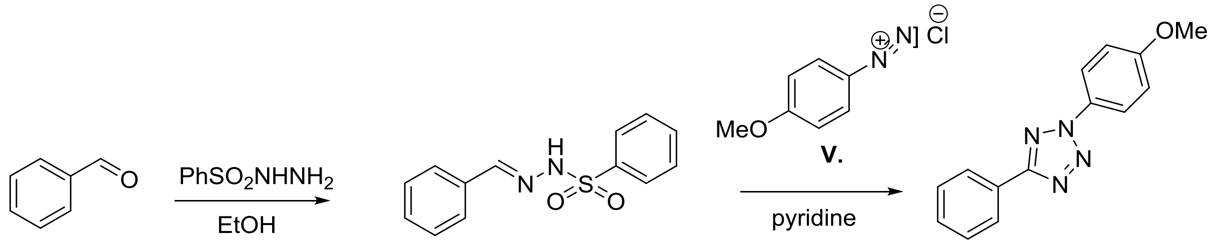

I. II. III.

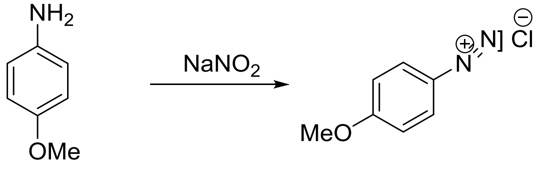

IV. V.

Supplementary Figure S9. Synthetic route for 2-(4-methoxyphenyl)-5-phenyl-2*H*-tetrazole (**III**).

5.1. The synthesis of phenylsulfonylhydrazone (II)

The stirred solution of the aldehyde (**I**, 18.9 mmol, 2 g) in ethanol (20 mL) was mixed with the solution of phenylsulfonylhydrazine (18.9 mmol, 3.3 g) in ethanol (20 mL). After stirring for 30 min at room temperature, the mixture was cooled and diluted with water (50 mL). The formed precipitate was filtered off, washed with hexane (3x10 mL) and dried.

5.2. The synthesis of 4-methoxyphenyldiazonium salt (V)

A cooled solution of sodium nitrite (7.7 mmol, 0.54 g) in water (4 mL) was added to a solution of aniline (**IV**, 7.7 mmol, 0.95 g) in 50% ethanol (13 mL) and concentrated hydrochloric acid (2 mL) below 5˚C.

5.3. The synthesis of 2-(4-methoxyphenyl)-5-phenyl-2*H*-tetrazole (III)

The solution of 4-methoxyphenyldiazonium salt (**V**) was added dropwise over a period of 30 min to a stirred solution of phenylsulfonylhydrazone (**II**, 7.7 mmol, 2 g) in pyridine (40 mL) at -10–-15˚C. The reaction mixture was extracted with chloroform (3x20 mL) and water (3x20 mL). The chloroform layer was extracted with dilute hydrochloric acid (1%, 3x20 mL), and water (3x20 mL). The chloroform layer was dried over anhydrous Na_2SO_4 and evaporated under reduced pressure. After removal of the solvent the resulting residue was purified by silica gel column chromatography using CH_2Cl_2 as eluent to give the corresponding 2-(4-methoxyphenyl)-5-phenyl-2*H*-tetrazole (**III**). The ^1H and ^{13}C NMR spectra of **III** were recorded in $CDCl_3$ and are in accordance with data from literature[1,2] (**Fig. S10, S11**). MS spectra and LC-MS analysis were recorded on Agilent 6410 Triple Quadrupole LC/MS mass spectrometry system. LC-MS measurements were performed using Phenomenex Kynetex 2,6µm C18, 100 Å, 50x21mm column, acetonitrile 70%, water (0.1% HCOOH) 30% as mobile phase at 0.3 ml/min flow rate. The MS detector was operated in positive/negative electrospray ionization mode, with source temperature of 350°C, capillary voltage 4000V, fragmentor 120V and with MS2Scan mode, at least +/- 50 amu around molecular ion (**Fig. S12, S13**).

^{1}H NMR (400 MHz, Chloroform-*d*) δ 8.17 (dd, *J* = 56.4, 7.8 Hz, 4H), 7.51 (d, *J* = 7.0 Hz, 3H), 7.05 (d, *J* = 8.5 Hz, 2H), 3.88 (s, 3H)

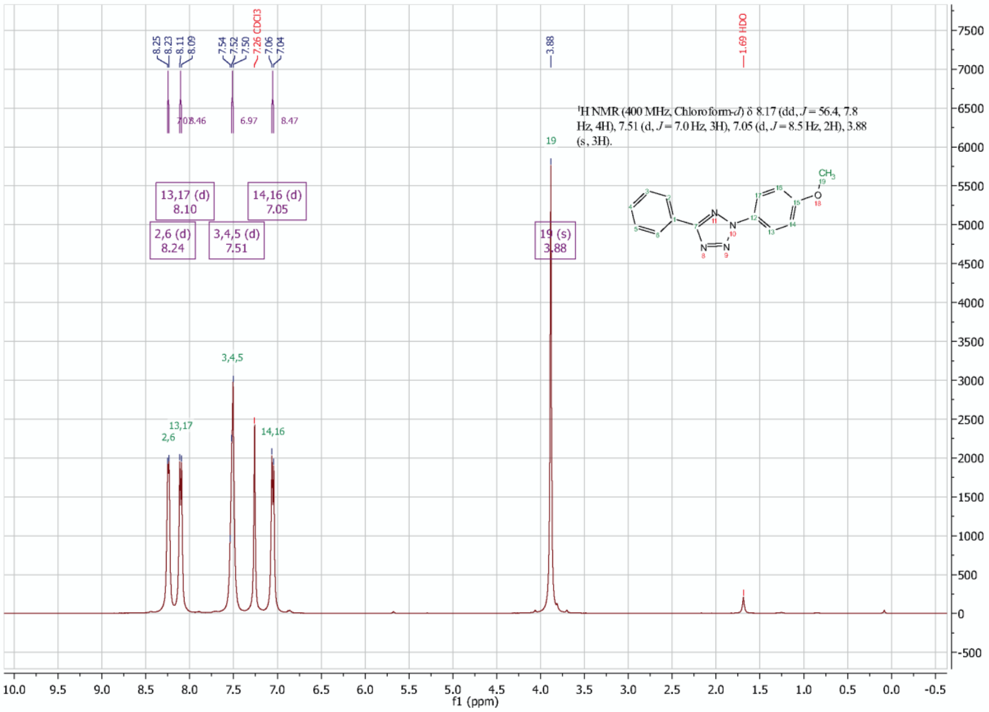

Supplementary Figure S10. The ^{1}H NMR spectra of the 2-(4-methoxyphenyl)-5-phenyl-2*H*-tetrazole (III).

^{13}C NMR (101 MHz, $CDCl_3$) δ 165.39, 160.89, 130.86, 129.35, 127.71, 127.41, 121.81, 115.08, 77.80, 77.68, 77.48, 77.16, 56.10.

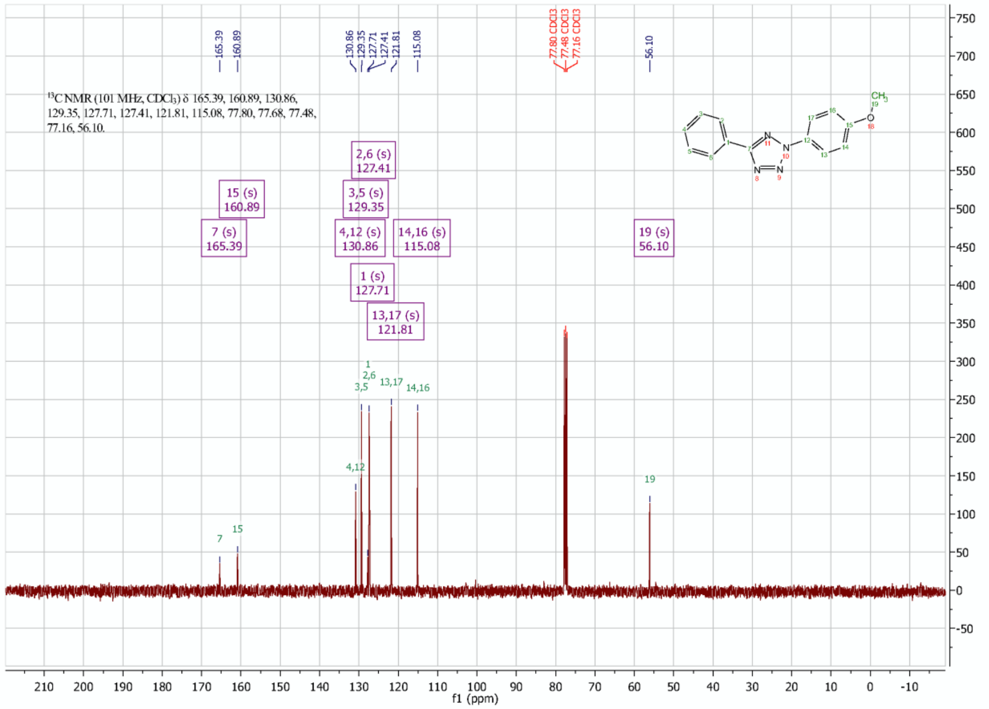

Supplementary Figure S11. The ^{13}C NMR spectra of the 2-(4-methoxyphenyl)-5-phenyl-2*H*-tetrazole (III).

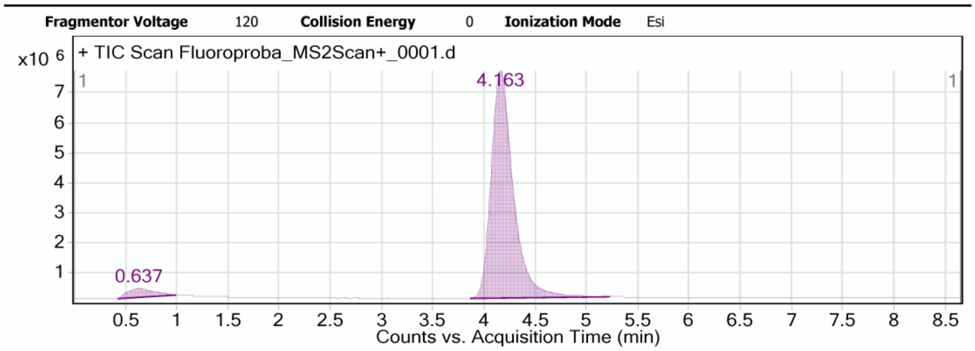

Integration Peak List

Peak	Start	RT	End	Height	Area	Area %
1	0.422	0.637	0.993	294766	5402096	4.74
2	3.865	4.163	5.231	7594319	113989807	100

Supplementary Figure S12. The LC chromatogram of the 2-(4-methoxyphenyl)-5-phenyl-2*H*-tetrazole (III).

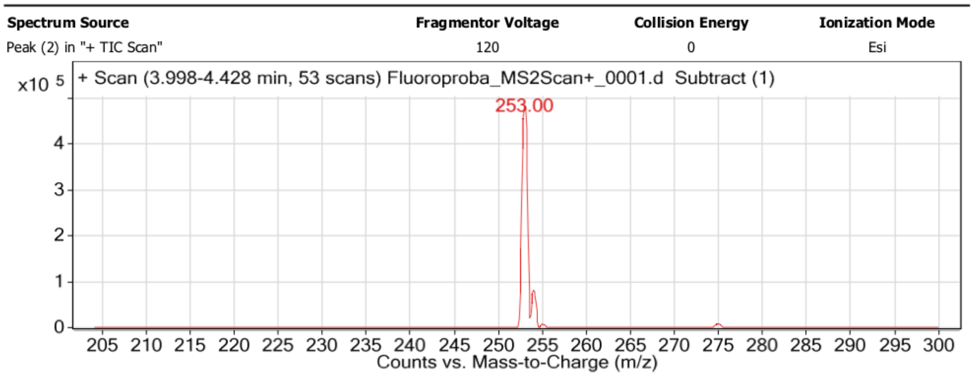

Supplementary Figure S13. The ESI-MS spectra of the compound with LC-retention time of 4.163 min corresponding to 2-(4-methoxyphenyl)-5-phenyl-2*H*-tetrazole (III).

6. The effect PAL- and FDC1- whole cell densities upon th e fluorescent signal intensities

Assay conditions: *Pc*PAL whole cells of OD_{600}~2 harvested from 400 µL culture medium were suspended in 400 µL (for final OD_{600}~1) or 200 µL (for final OD_{600}~2) substrate solution (L-Phe 2 mM) in Tris buffer (20 mM Tris, 100 mM NaCl, pH 8), followed by the addition of FDC1 cells resuspended in 400 µL (for final OD_{600}~1) or 200 µL (for final OD_{600}~2) Tris buffer. The reactions with a final volume of 800 µL (for reactions with final OD_{600}~1) or 400 µL (for reactions with final OD_{600}~2) were incubated overnight at 30 °C and 200 rpm. Further, extractions with 800 µL (for reactions with final OD_{600}~1) or 400 µL (for reactions with final OD_{600}~2) *n*-hexane were performed, followed by centrifugation at 4000 rpm, 4 °C for 20 min. 175 µL of extract was moved into a Corning 96-well Clear Flat Bottom UV-transparent microplate and mixed with 25 µL of diaryltetrazole **4** 1 mg/mL solution in *n*-octane. The UV reaction was carried out at 302 nm for 1 min using a handheld UV lamp. 100 µL from the samples were moved into Corning 96-well Black Flat Bottom plates and the fluorescence measurement was performed setting the excitation wavelength at 360 nm and the emission at 460 nm using TECAN Spark 10M microplate reader. Two negative controls (NC samples), one for final OD_{600}~1 and one for final OD_{600}~2, were prepared identical as the reactions but omitting the use of substrate, L-Phe. The blank sample (the background) consisted of 175 µL of *n*-hexane mixed with 25 µL of diaryltetrazole probe **4** (prepared as previously described). The negative controls and the blank sample were irradiated and measured as described above. All experiments were performed in triplicate and standard deviations from mean values are given.

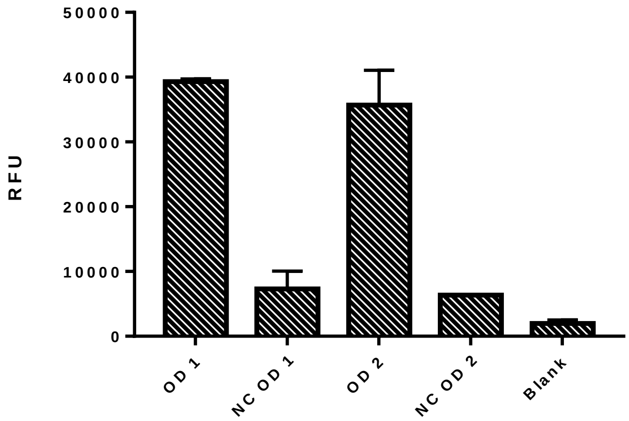

Supplementary Figure S14. Fluorescent enzyme-coupled assay using two different (but equal related to each other) cell densities for *Pc*PAL and FDC1 whole cell-biocatalysts.

7. Method validation

Assay conditions: The assay was performed under the optimized conditions described in the Materials and methods of the main manuscript. Whole cell-biocatalysts (*At*PAL, Duet-*At*PAL-FDC1, *Pf*HAL, *Rt*PAL) were obtained similarly as *Pc*PAL whole cells, described in the main manuscript, section Materials and methods. The substrate (L-Phe, L-Tyr) concentration was in all cases 2 mM. All experiments were performed in triplicate and standard deviations from mean values are given.

a) **b)**

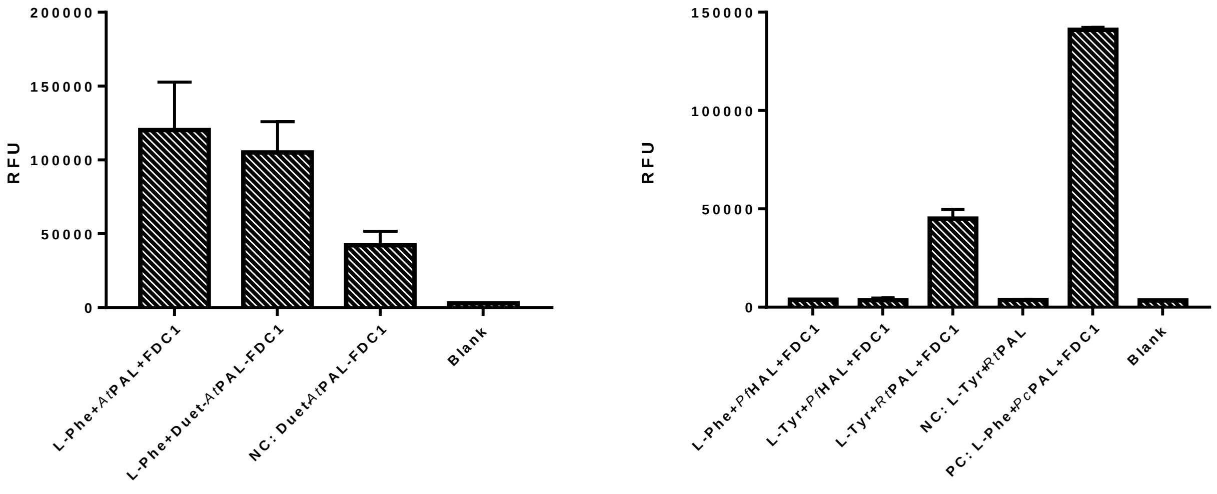

Supplementary Figure S15. Fluorescent enzyme-coupled assay using **a)** PAL from *Arabidopsis thaliana* (*At*PAL) coupled with FDC1 in both individual and combined whole cell-biocatalysts forms and **b)** PAL from *Rhodotorula toruloides* (*Rt*PAL) for TAL-activity detection (NC: negative control without FDC1 cells; PC: positive control).

8. Construction of pcpal-fdc1-pCDFDuet-1 expression vector

The pCDFDuet-1 vector containing two multiple cloning sites (MCS) was employed for the molecular cloning of both *Sc*FDC1 and *Pc*PAL. The gene of *Sc*FDC1, obtained through digestion of pTfdc1Sc plasmid[3] with restriction enzymes SalI and HindIII, together with SalI and HindIII digested pCDFDuet-1 vector were extracted from the agarose gel and ligated (1 h incubation at 22 °C). 5 µL from the ligation reaction were transformed through heat-shock into *E. coli* XL-1 Blue competent cells, the grown colonies were selected and the presence of the insert (*fdc1* gene) was verified through colony PCR (**Fig. S16b**). The primers used for colony PCR are: T7_for: 5'AATACGACTCACTATAGGGGAATTG3' and Duet_DOWN1: 5'GATTATGCGGCCGTGTACAA3'. From one of the positive colonies an overnight culture was grown (5 mL sterile LB supplemented with tetracycline 12.5 µg/mL and streptomycin 30 µg/mL) at 37 °C and 180 rpm and the plasmid fdc1-pCDFDuet-1 was extracted.

Next *pcpal* gene was cloned into MCS-2 of the vector already containing *fdc1* gene in MCS-1 (fdc1-pCDFDuet-1). First *pcpal* gene was amplified by PCR, using the pcpal-pET19b plasmid[4] as template, and restriction sites for NdeI and AatII were introduced. The primers used for the PCR experiments are NdeI_for: 5'ATTCATATGATGGAAAACGGAAACGGAGC3'

and AatII_rev: 5'AATGACGTCTCAGGAAATGGGCAAGGG3'.

The purified PCR product and fdc1-pCDFDuet-1 plasmid were digested with NdeI and AatII restriction enzymes, extracted from the agarose gel and then ligated as previously described. After transformation through heat-shock of the ligation mixture into *E. coli* XL-1 Blue competent cells, the presence of the insert (*pcpal* gene) in the grown colonies was tested through colony PCR using the primers: Duet_UP2: 5'TTGTACACGGCCGCATAATC3' and T7_rev: 5'TGCTAGTTATTGCTCAGCGG3'. One of the positive colonies was used to inoculate an overnight culture (5 mL sterile LB supplemented with tetracycline 12.5 µg/mL and streptomycin 30 µg/mL) at 37 °C and 180 rpm and the plasmid pcpal-fdc1-pCDFDuet-1 was extracted.

a) b) 1 2 3 4 5 6 7 8 9 10 11

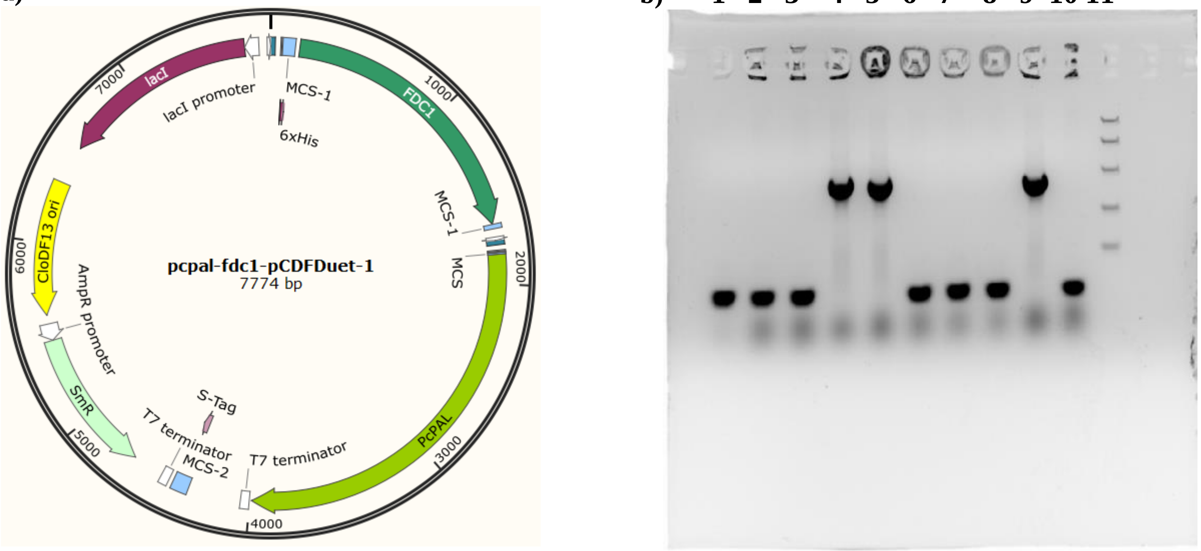

Supplementary Figure S16. a) Snapgene map of FDC1-*Pc*PAL plasmid construct and **b)** full-length agarose gel for colony PCR confirming the presence of *fdc1* gene (1756 bp) in pCDFDuet-1 vector in colonies 4, 5 and 9 (**1-10**: colonies, **11**: DNA ladder: 10000, 4000, 2000, 1000, 500 bp).

9. References

1. Ito, S., Tanaka, Y., Kakehi, A., Kondo, K. A Facile Synthesis of 2,5-Disubstituted Tetrazoles by the Reaction of Phenylsulfonylhydrazones with Arenediazonium Salts. *Bull. Chem. Soc. Jpn.* **49**, 1920-1923, doi:10.1246/bcsj.49.1920 (1976).

2. Wang, Y., Song, W., Hu, W. J. & Lin, Q. Fast alkene functionalization in vivo by photoclick chemistry: HOMO lifting of nitrile imine dipoles. *Angew. Chem. Int. Ed.* **48**, 5330-5333, doi:10.1002/anie.200901220 (2009).

3. McKenna, R. & Nielsen, D. R. Styrene biosynthesis from glucose by engineered *E. coli*. *Metab. Eng.* **13**, 544-554, doi:10.1016/j.ymben.2011.06.005 (2011).

4. Dima, N. A. *et al.* Expression and purification of recombinant phenylalanine ammonia-lyase from *Petroselinum crispum*. *Stud. Univ. Babes-Bolyai Chem.* **61**, 21-34 (2016).
